# Previously Untreated Ulcerative Colitis With Newly Diagnosed Primary Sclerosing Cholangitis: A Case Report

**DOI:** 10.7759/cureus.45311

**Published:** 2023-09-15

**Authors:** Rahel Tekeste, Elvia Villarreal, Matthan Moy, Bernard Karnath

**Affiliations:** 1 Internal Medicine, University of Texas Medical Branch at Galveston, Galveston, USA

**Keywords:** jaundice cholestatic, endoscopic retrograde cholangiopancreatography (ercp), magnetic resonance cholangiopancreatography (mrcp), lack of health insurance, social determinants of health (sdoh), inflammatory bowel disease, ulcerative colitis (uc), primary sclerosing cholangitis (psc)

## Abstract

Primary sclerosing cholangitis (PSC) is a rare type of autoimmune hepatic disease with unknown pathophysiology, often a sequela of ulcerative colitis (UC). Liver transplant, though curative, is inaccessible to many patients due to stringent organ availability and barriers to sufficient insurance coverage. Low health literacy and low socioeconomic class can significantly limit healthcare access and thus worsen overall healthcare outcomes. Here, we present the case of an uninsured 49-year-old man with untreated UC who was diagnosed with PSC and subsequently became lost to follow-up. It is critical for providers to identify barriers to acquiring appropriate medical care, such as financial instability and low health literacy, when identifying and treating conditions like PSC. Moreover, more research should be performed to investigate alternative treatments for PSC.

## Introduction

Primary sclerosing cholangitis (PSC) is a rare type of autoimmune liver disease characterized by intrahepatic and/or extrahepatic biliary ductal strictures resulting in hepatobiliary destruction and an increased risk of hepatic cirrhosis, cholangiocarcinoma, and colorectal carcinoma [1].   

While much research has been dedicated to treatments of PSC, a quick PubMed search did not reveal case reports studying the role of financial stability and health literacy in the presentation and treatment of PSC. Here, we report a case of a newly diagnosed PSC in an uninsured 49-year-old male with past medical history of untreated ulcerative colitis (UC). The aim of this case report is to increase awareness of PSC, especially when complicated by social determinants of health.

## Case presentation

A 49-year-old man with past medical history of untreated UC presented to the emergency room (ER) with a chief complaint of right-upper-quadrant abdominal pain and scleral icterus. The patient stated that a week earlier, he had presented to an outside ER with right-upper-quadrant abdominal pain following a week-long alcohol binge. Laboratory results revealed mildly elevated liver enzymes. He was diagnosed with “food poisoning” and discharged with unknown pain medication. His abdominal pain eventually worsened and was accompanied by scleral icterus. This led him to present at our institution. 

The patient stated that he was first diagnosed with UC in 2001. He received an unknown medication regimen at an outside hospital. He did not recall which provider saw him, so we were unable to request medical records. He reported that his last colonoscopy was in 2003, showing “advanced UC.” He was recommended to either continue the unknown medical therapy or consent to a colectomy. He declined both options due to a reported lack of perceived benefit from medications and an inability to take off work for surgery. He had not seen any medical provider since then. He reported multiple episodes of watery diarrhea per day. He denied any recent acetaminophen or illicit drug use. 

Physical examination revealed diffuse abdominal tenderness to palpation, mild jaundice, and scleral icterus. He was hemodynamically stable and afebrile. Laboratory results showed increased total, unconjugated, and conjugated bilirubin, alkaline phosphatase, aspartate transaminase (AST), alanine transaminase (ALT), and gamma-glutamyl transferase (GGT) (Table 1). The following were within normal limits: serum alcohol; hepatitis A, B, and C serologies; anti-mitochondrial antibody (AMA), liver-kidney microsomal antibody (LKM), alpha 1 antitrypsin, and ceruloplasmin. Immunoglobulin G4 (IgG4) was ordered but later cancelled because the patient was discharged before the lab sample could be taken. 

**Table 1:** Laboratory data

Laboratory test	Value at admission	Value at outpatient follow-up	Reference range
Total bilirubin	10.3 mg/dl	1.0 mg/dL	0.1-1.1 mg/dl
Unconjugated bilirubin	1.8 mg/dl	0.2 mg/dL	0.1-1.1 mg/dl
Conjugated bilirubin	5.7 mg/dl	0.0 mg/dL	0.0-0.3 mg/dl
Alkaline phosphatase	382 U/L	137 U/L	34-122 U/L
aspartate aminotransferase (AST)	521 U/L	45 U/L	13-40 U/L
alanine transaminase (ALT)	399 U/L	56 U/L	5-50 U/L
Total protein	8.1 g/dL	7.5 g/dL	6.3-8.2 g/dL
Albumin	4.4 g/dL	4.3 g/dL	3.5-5.0 g/dL
Gamma-glutamyl transferase (GGT)	505 U/L		13-58 U/L
Serum alcohol	<10 mg/dL		Negative
Hepatitis A virus antibody IgM	Negative		Negative
Hepatitis B surface antibody	Negative		Negative
Hepatitis B surface antigen	Negative		Negative
Hepatitis B core IgM antibody	Negative		Negative
Hepatitis C quantitative interpretation	Not detected		Negative
Anti-mitochondrial antibody (AMA)	4.5 U		0.0-24.9 U
Liver-kidney-microsome antibodies (LKM), IgG	<1:20		Less than 1:20 normal
Alpha 1 antitrypsin	173 mg/dL		83-199 mg/dL
Ceruloplasmin	48 mg/dL		25-63 mg/dL

**Figure 1:**
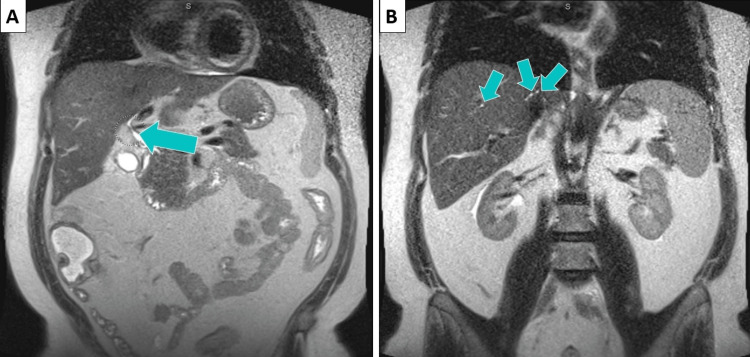
MRCP results: blue arrows point to the extrahepatic (A) and intrahepatic (B) bile duct strictures. MRCP: magnetic resonance cholangiopancreatography

Abdominal ultrasound revealed a hydropic gallbladder without acute cholecystitis and grade I diffuse hepatic steatosis. Magnetic resonance imaging (MRI)/magnetic resonance cholangiopancreatography (MRCP) showed chronic inflammation throughout the colon and irregularity of the intrahepatic and extrahepatic bile ducts, suggesting PSC. There was no evidence of cirrhosis or focal liver lesions and no cholelithiasis or choledocholithiasis (Figure 1). A biopsy was not performed during this admission. He was diagnosed with PSC. The gastroenterology consult team recommended a liver transplant, although this was not possible due to the patient’s lack of health insurance. Case management helped the patient set up an insurance plan. He was discharged with plans to follow up with gastroenterology and general medicine.

In a general medicine follow-up appointment at our institution’s free health clinic a few weeks later, a repeat hepatic function panel showed decreased total, unconjugated, and conjugated bilirubin, alkaline phosphatase, ALT, and AST (Table 1). Total protein and albumin remained within normal limits. He was started on mesalamine suppository for his UC. Unfortunately, he did not attend gastroenterology clinic follow-up at our institution. At the time of publishing, the patient has not attended any other clinic appointments per the University of Texas Medical Branch (UTMB)’s electronic medical records.  

We called and emailed the patient multiple times to ask for consent for this report, but he did not respond. No patient-identifiable information is included in this case report.

## Discussion

PSC is a rare, but clinically relevant, sequelae of UC. The pathophysiology of PSC is currently unknown [1]. As of 2017, the prevalence of PSC in the United States is 0.4-2.0 per 100,000 every year [1]. Studies have shown that there is a strong association between UC and PSC, with about 80% of patients with PSC having underlying UC [2]. It has been found that about 5% of patients with UC develop PSC, which is why annual liver function studies are often recommended for patients with UC to screen for PSC [3]. Most patients upon diagnosis are males aged 30-40 years old with a history of inflammatory bowel disease (IBD) [1].  

The diagnostic criteria of PSC include elevated serum alkaline phosphatase and bilirubin, along with the exclusion of other causes of cholestasis, such as primary biliary cholangitis, choledocholithiasis, biliary strictures, and pancreatic neoplasm [4]. MRCP or endoscopic retrograde cholangiopancreatography (ERCP) may reveal intrahepatic and extrahepatic bile duct strictures [4]. MRCP is preferred over ERCP due to increased sensitivity/specificity and decreased risk of complications, including pancreatitis [1,4,5]. 

The only curative treatment for PSC is a liver transplant. However, transplant approval is limited by organ access, insurance coverage, and cost [6]. In addition, there may be other contraindications to liver transplant, such as substance abuse and insufficient social support [6]. The exact cost of a hepatic transplant in the United States can vary based on the source. For instance, a 2023 article found that the cost of inpatient hospitalizations for patients receiving liver transplants from 2016 to 2019 was approximately $145,000 [7]. Medications, such as ursodeoxycholic acid (UDCA), have also been proposed, although the British Society of Gastroenterology/UK-PSC guidelines, American College of Gastroenterology (ACG), and Journal of Hepatology have not sufficiently proven clinical utility [1,5,8]. The standard dose of UDCA per the ACG is 13-15 mg/kg/day [5]. Our patient’s most recent recorded weight is 104.3 kg. Based on the unit price provided by the 2023 National Average Drug Acquisition Cost of the United States Centers for Medicare & Medicaid Services, a 30-day supply of ursodiol 300 mg for our patient would cost approximately $75 [9]. 

This case exemplifies a typical presentation of PSC in the presence of UC based on gender and age [6]. Initial presentation can be asymptomatic for prolonged periods of time. It can also present with biliary tract obstruction, as in our patient, or it may present with cholangitis [6,10]. Like most patients with PSC, ours was diagnosed with MRCP. Due to the presence of scleral icterus and recent history of binge drinking, alcoholic hepatitis was an important differential. However, in our patient, the elevation of AST and ALT was higher than expected for alcoholic hepatitis, and the AST:ALT ratio was less than 1. 

Our patient’s financial status and health literacy are pertinent to understanding his medical history and presentation to our institution. The patient reported that he had not been compliant with his UC medications and did not follow his doctor’s advice on the importance of treating his UC as he believed he did not need the medications. These are examples of low health literacy. Patients with low health literacy are more likely to have a poor understanding of their conditions and medications [11]. They are also more likely to communicate passively with their physicians [11]. These factors can strain the patient-physician relationship and thus impair patient health outcomes [11]. Clinicians can address low health literacy in the outpatient setting by establishing an effective relationship with patients. This can be done through proper communication of health information and the identification of potential social determinants of health that may influence treatment adherence. This relationship may lead to improved health outcomes and decrease the risk of clinician burnout [11]. 

In addition, our patient’s unemployed and uninsured status likely significantly contributed to his inability to afford continual medical treatment, which consequently worsened his UC and increased the likelihood of him developing PSC. Clinicians can help dispense insurance information by collaborating with social workers and case management. In addition, it may be helpful to refer such patients to free clinics if possible to avoid non-adherence due to financial constraints. 

Ultimately, because we were unable to contact the patient, we can only estimate what factors influenced his continued medical noncompliance after discharge. Regardless, we believe that incorporating social determinants of health in treatment plans is pertinent to provide well-rounded clinical care.

## Conclusions

PSC is a rare but critical differential to consider when exploring causes of cholestasis, especially in the presence of ulcerative colitis, due to its devastating sequelae. Liver transplant, though effective, is inaccessible to many patients due to stringent organ availability and barriers to sufficient insurance coverage. It is critical for providers to identify barriers to acquiring appropriate medical care, such as financial instability and low health literacy, when identifying and treating conditions like PSC. Moreover, more research should be done to investigate alternative treatments for PSC.
